# Advances in Humanized Mouse Models to Improve Understanding of HIV-1 Pathogenesis and Immune Responses

**DOI:** 10.3389/fimmu.2020.617516

**Published:** 2021-03-05

**Authors:** Amy Gillgrass, Jocelyn M. Wessels, Jack X. Yang, Charu Kaushic

**Affiliations:** ^1^ Department of Medicine, McMaster University, Hamilton, ON, Canada; ^2^ McMaster Immunology Research Centre, McMaster University, Hamilton, ON, Canada; ^3^ Michael G. DeGroote Institute for Infectious Disease Research, McMaster University, Hamilton, ON, Canada; ^4^ Department of Obstetrics and Gynecology, McMaster University, Hamilton, ON, Canada

**Keywords:** HIV-1, humanized mouse, pathogenesis, immune response, mucosal infection, microbiota, co-infection, vaccines

## Abstract

Although antiretroviral therapy has transformed human immunodeficiency virus-type 1 (HIV-1) from a deadly infection into a chronic disease, it does not clear the viral reservoir, leaving HIV-1 as an uncurable infection. Currently, 1.2 million new HIV-1 infections occur globally each year, with little decrease over many years. Therefore, additional research is required to advance the current state of HIV management, find potential therapeutic strategies, and further understand the mechanisms of HIV pathogenesis and prevention strategies. Non-human primates (NHP) have been used extensively in HIV research and have provided critical advances within the field, but there are several issues that limit their use. Humanized mouse (Hu-mouse) models, or immunodeficient mice engrafted with human immune cells and/or tissues, provide a cost-effective and practical approach to create models for HIV research. Hu-mice closely parallel multiple aspects of human HIV infection and disease progression. Here, we highlight how innovations in Hu-mouse models have advanced HIV-1 research in the past decade. We discuss the effect of different background strains of mice, of modifications on the reconstitution of the immune cells, and the pros and cons of different human cells and/or tissue engraftment methods, on the ability to examine HIV-1 infection and immune response. Finally, we consider the newest advances in the Hu-mouse models and their potential to advance research in emerging areas of mucosal infections, understand the role of microbiota and the complex issues in HIV-TB co-infection. These innovations in Hu-mouse models hold the potential to significantly enhance mechanistic research to develop novel strategies for HIV prevention and therapeutics.

## Introduction

Currently approximately 38 million people are living with human immunodeficiency virus-type 1 (HIV-1), the underlying cause of acquired immune deficiency syndrome (AIDS) (1). Although treatment with antiretroviral therapy (ART) has transformed HIV from a deadly infection into a chronic disease, it does not clear the latent viral reservoir, therefore there is still no cure for HIV infection (2). Furthermore, even with ART, HIV infection increases risks of co-infection with other pathogens such as *Mycobacterium tuberculosis* (*Mtb*) (3). Additional research is required to advance the current state of HIV management and potential therapeutic strategies, in addition to understanding mechanisms of HIV pathogenesis. Although animal models such as non-human primates (NHP) have been used extensively in HIV research and have provided critical advances in knowledge within the field, there are several issues including host-restriction factors, ethics, and cost that can limit their use (4, 5). Furthermore, the human species-specific tropism of HIV-1 has prevented the use of traditional murine models leading to a lack of small animal models for *in vivo* HIV-1 research (6).

Humanized mouse (Hu-mouse) models, or immunodeficient mice engrafted with human immune cells and/or tissues, provide a cost-effective and practical approach to creating models for HIV-1 research. Unlike traditional mouse models, Hu-mouse models effectively sustain HIV-1 infections while also recapitulating relatively accurate *in vivo* immune responses to the infection due to the reconstitution with human immune cells when compared to other animal models (7). This review will outline the advances in Hu-mouse models that have made them useful in HIV-1 research and a convenient alternate to NHP. Furthermore, numerous novel modifications of Hu-mice demonstrate potential to advance knowledge in virus transmission, infection, evolution, pathogenesis, prevention, latency, cure, and disease interaction such as *Mtb* co-infection. Additionally, since the major physiological route of HIV-1 transmission in humans is by the mucosal route (intrarectally or intravaginally) (8), this review will detail the use of Hu-mice in elucidating mechanisms involving mucosal infections and discuss how microbiota may be involved.

## Hu-Mouse Models for HIV-1 Research

Currently, some of the most widely used Hu-mouse models in HIV research take advantage of three major immunocompromised features which allow for the successful engraftment of human cells or tissues. NOD.Cg-Prkdc^scid^Il2rg^tm1Sug^ (NOG) (9), NOD.Cg-Prkdc^scid^Il2rg^tm1Wjl^ (NSG) (10, 11), and NOD.Cg-Rag1^tm1MoM^Il2rg^tm1Wjl^ (NRG) (12) are on the non-obese diabetic (NOD) background that leads to suppressed mouse macrophage phagocytic activity. Mice with the *Prkdc* ^scid^ or Rag1/Rag2 loci mutation lack mature T and B lymphocytes while the *Il2rg* gene mutation effectively eliminates mouse NK cell activity (13). The most common engraftment method of human cells is the intravenous or intrahepatic injection of CD34+ hematopoietic stem cells (14) into adult or newborn immunodeficient mice, respectively, after myeloablative irradiation or administration of myeloablative doses of drugs such as busulfan (15). This engraftment method has been performed in each model (NOG, NSG, NRG) yielding reconstitution of human CD4+ and CD8+ T cells, monocytes, macrophages, dendritic cells (DCs) and progenitor B cells in peripheral blood, primary and secondary lymphoid tissues (12, 16).

The unique engraftment method using surgical implantation of human fetal liver and thymus tissues followed by injection of matched CD34+ hematopoietic stem cells (HSCs) gave rise to the bone marrow liver thymus (BLT) model (17–19). The human thymic tissue allows for T cell education in the context of human cells (20). Both HSC-only and BLT methods are able to successfully reconstitute human monocytes, dendritic cells, T cells, and B cells in peripheral blood and tissues, but higher cell counts were observed in the BLT engraftment method (21, 22). The HSC-only method demonstrated better human B cell and myeloid cell development (21) while additional thymus support yielded higher CD3^+^ T cell reconstitution in the spleen (21), gastrointestinal (GI) (22) and gut-associated lymphoid (GALT) tissues (18, 21, 23) (**Table 1**). Both methods have demonstrated similar susceptibility to HIV infection, trends in CD4+ T cell depletion, and persistent viral reservoirs *in vivo* (21). The major difference between the two methods is that BLT-engrafted mice have measurable T cell response against HIV-1 because the human thymic tissue allows the resulting T cells to respond to the HIV-1 antigen presentation by human leukocyte antigen (HLA) generating HLA-restricted anti-HIV-1 human T cell response, which is absent in the current HSC-only method (18, 48) (**Table 1**). This has led to the BLT model being the current gold standard for studying HIV-1 immune responses (17, 49, 50).

**Table 1:** Summary of reported reconstitution of major human immune cell types within current generation and next generation hu-mice for HIV studies.

Humanized Mouse Model	Human immune cell reconstitution	References
**Current Generation Models**		
HSC-DKO/BRG	**PB:** CD45+ lymphocytes, CD4+ T cells, CD8+ T cells **BM:** CD45+ lymphocytes, mature and immature B cells **LT:** CD45+ lymphocytes, CD3+ T cells, CD4+ T cells, CD8+ T cells, T regulatory cells, mature and immature B cells	(24–27)
HSC-NOG	**PB:** CD45+ lymphocytes, CD3+ T cells, immature B cells **BM:** CD45+ lymphocytes, immature B cells **LT:** CD45+ lymphocytes, CD4+ T cells, CD8+ T cells, immature B cells	(16, 28)
HSC-NSG HSC-NRG	**PB:** CD3+ T cells, immature B cells **BM:** CD45+ lymphocytes, mature and immature B cells, immature NK cells **LT:** CD4+ T cells, CD8+ T cells, mature and immature B cells **FRT:** CD45+ cells, CD4+ T cells, CD68+ macrophages	(12, 29–31)
NSG-BLT*	**PB:** CD3+ T cells, CD4+ T cells, CD8+ T cells, immature B cells **BM:** CD45+ lymphocytes, mature and immature B cells **LT:** CD4+ T cells, CD8+ T cells, mature and immature B cells **GI:** CD45+ lymphocytes, CD4+ T cells, CD8+ T cells, B cells, CD68+ macrophages, dendritic cells **FRT:** CD3+ T cells, CD4+ T cells, CD68+ macrophages, CD11c+ dendritic cells	(18, 32, 33)
**Next Generation Models**		
HSC-DRAG* HSC-DRAGA*	**PB:** CD3+ T cells, CD4+ T cells, CD8+ T cells, isotype switched mature B cells **BM:** CD45+ lymphocytes **LT:** CD45+ lymphocytes, CD4+ T cells, CD8+ T cells, T regulatory cells, dendritic cells **GI:** CD45+ lymphocytes, CD4+ T cells, CD8+ T cells, naïve and memory B cells **FRT:** CD4+ T cells, T follicular helper cells, naïve and memory B cells	(34–36)
HSC-BRGST HSC-BRGSA2DR2*	**PB:** CD45+ lymphocytes, CD3+ T cells, CD4+ T cells, CD8+ T cells, T follicular helper cells (HSC-BRGST only), isotype switched mature B cells **BM:** CD45+ lymphocytes, isotype switched B cells **LT:** CD45+ lymphocytes, CD4+ T cells, CD8+ T cells, isotype switched mature B cells, T follicular helper cells (HSC-BRGST only), central and effector memory T cells	(37, 38)
NSGW-NeoThy	**PB:** CD45+ lymphocytes, CD3+ T cells, CD4+ T cells, CD8+ T cells, regulatory T cells, B cells, monocytes/macrophages **BM:** CD45+ lymphocytes, CD3+ T cells, B cells **LT:** CD45+ lymphocytes, CD3+ T cells, regulatory T cells, B cells, monocytes/macrophages	(39)
HSC-NOG-EXL	**PB:** CD4+ T cells, CD8+ T cells, CD33+ myeloid cells, basophils, neutrophils, NK cells, monocytes, dendritic cells **BM:** CD3+ T cells, B cells, mast cells, basophils **LT:** CD3+ T cells, B cells, mast cells, basophils, dendritic cells **GI:** Mast cells, basophils	(40, 41)
HSC-NSGS/NSG-SGM3	**PB:** CD4+ T cells, B cells, T regulatory cells **BM:** CD3+ T cells, CD4+ T cells, T regulatory cells, B cells, dendritic cells **LT:** CD3+ T cells, CD4+ T cells, T regulatory cells, B cells, CD33+ myeloid cells	(42–44)
HSC-SRG-15	**PB:** CD45+ lymphocytes, mature NK cells **BM:** mature NK cells, CD45+ lymphocytes, CD3+ T cells, CD4+ T cells, CD8+ T cells **LT:** tissue-resident NK cells, CD45+ lymphocytes, CD3+ T cells, CD4+ T cells, CD8+ T cells	(31)
HSC-NSG-15	**PB:** mature NK cells, CD3+ T cells **BM:** mature NK cells, CD45+ lymphocytes, CD3+ T cells, CD4+ T cells, CD8+ T cells, B cells **LT:** mature NK cells, CD45+ lymphocytes, CD3+ T cells, CD4+ T cells, CD8+ T cells, B cells	(45)
HSC-MITRG HSC-MISTRG	**PB:** Monocytes, functional NK cells, CD45+ lymphocytes, CD3+ T cells, naïve CD4+ T cell, naïve CD8+ T cell, immature B cells **BM:** CD45+ lymphocytes, CD33+ myeloid cells, functional monocytes **LT:** functional NK cells, monocytes, dendritic cells **GI:** CD68+ myeloid cells	(46, 47)

PB, Peripheral blood; BM, Bone marrow; LT, Primary and secondary lymphoid tissue; GI, Gastrointestinal organs; FRT, Female reproductive tract.

*HLA-restricted immune responses observed.

## HSC Engraftment Models (Current Generation): NOG, NSG, NRG, DKO/BRG, NSG-BLT

### NOG and NSG

The NOG and NSG mice differ in the IL-2 receptor gene (Il2rg) which is truncated in NOG and knocked-out in NSG. Both humanized NOG (hu-NOG) and humanized NSG (hu-NSG) mice have demonstrated successful engraftment of HSC with substantial human lymphoid repopulation (29, 51–53). Intraperitoneal and intravenous routes of HIV-1 infection into both types of mice demonstrated viremia and viral dissemination throughout lymphoid tissues (29, 51–54).

Hu-NOG mice have furthered the understanding of HIV-1 transmission as well as treatment testing and development. These mice can generate B cells that secrete isotype-switched, HIV-specific IgG antibodies (16). Studies using hu-NOG models investigated the role of human anti-viral factors in human transmission of HIV-1 (55) and treatment options with novel therapeutics such as zinc-finger nucleases (ZFN) showing reduced viral loads and increased CD4+ T cell counts (56). Additionally, viral evolution and replication kinetics have been investigated using this model using various HIV-1 strains (57). Finally, hu-NOG mice were also used to investigate the efficacy of ART, long-acting antiretroviral compounds that showed reduced viral load and recovery of CD4+ T cell counts, and a latent viral reservoir with T cell depletion after treatment was stopped, similar to that seen in humans (58).

Numerous HIV-1 treatment methods have been tested on hu-NSG mice including combination ART (cART) (29, 59), highly active antiretroviral therapy (HAART), and neutralizing antibody treatment (54). Similar to the response seen in hu-NOG mice, latent infection was established and persisted during treatment (29, 59). Resting memory CD4+ T cells were the major viral reservoir (29), unaffected by the length of cART treatment (59). A recent study using hu-NSG mice showed that HIV-1 hematopoietic stem/progenitor cell-based gene therapy targeting CCR5 and HIV-1 LTR could be used as anti-HIV strategy (60). Another study tested long-acting, slow-release antiviral therapy in combination with CRISPR-Cas9 gene editing to eliminate latent HIV-1 in Hu-mice, and was the first to demonstrate that permanent viral elimination is possible (61). Additionally, the hu-NSG model has been used to provide better understanding of HIV-1 pathogenesis. The model revealed that cell-to-cell viral transmission efficiently disseminated infection within tissues, suggesting anatomically localized spread would be an area of future investigation for targeted treatments (62). HIV-1 disease progression was also investigated in hu-NSG mice by tracking viral seeding into different tissue compartments providing a picture of the HIV-1 infection timeline (63). Although it has been demonstrated that hu-NSG mice successfully reconstitute human CCR5+ CD4+ T cells within the female reproductive tract (FRT) (29, 30), to date, HSC-engrafted NSG mice have not been utilized widely to study mucosal and sexual transmission of HIV-1 (30).

### NRG and DKO (BRG)

Like the NSG model, the more radioresistant hu-NRG have similar successful engraftment of human peripheral blood mononuclear cells (PBMCs) or HSCs (12). HSC-engrafted NRG mice demonstrated successful mucosal HIV-1 challenge with viral dissemination throughout the FRT and lymphoid tissues (64, 65). An older, yet similar model without the NOD background termed Rag1^null^Il2rg^null^ or Rag2^null^Il2rg^null^ (DKO) mice (also known as BRG mice) (11, 66), also demonstrated susceptibility to both R5- and X4-tropic variants of HIV-1 *via* vaginal and rectal mucosal transmission with insights on therapy efficacy, latency and chronic infection (24–26, 67, 68). Furthermore, hu-DKO/hu-BRG mice have greatly contributed to cross-species transmission and viral evolution investigations (69, 70), as well as the development of Hu-mice based viral outgrowth assays to further the understanding of HIV latency (71, 72). Successful mucosal infection in hu-DKO and hu-NRG mice best models natural human routes of HIV-1 transmission and allows studies of microbiota alteration (65) and topical pre-exposure prophylaxis (PrEP) (67, 73–76).

Studies using hu-NRG mice investigated the role of plasmacytoid dendritic cells during infection (77) and HIV-1 latency, and revealed the persistence of type 1 interferon (IFN) signaling after cART treatment (78). Furthermore, therapeutics that enhance ART treatment such as broadly neutralizing antibodies (79) showed promise in this model for prevention of cell-to-cell HIV-1 transmission (80, 81). Novel CRISPR/CAS9 genome editing technology was used in PBMC-engrafted NRG mice and demonstrated excision of HIV-1 pro-viral DNA which reduced levels of HIV-1 (82). Additionally, single-cell RNA-sequencing was used in this model to characterize human innate immune cells in lymphoid tissues (83). Interestingly, despite the lack of isotype-switched mature B cells, hu-NRGs can still be a useful tool for certain vaccine investigations (84).

### NSG-BLT Engraftment Model

Compared to HSC-engrafted DKO, NSG, and NRG, the BLT engrafted NSG (NSG-BLT) hu-mice have the best overall reconstitution and functional human immune system for studying immune responses to HIV-1 infection (18, 49, 85, 86). For this reason, the BLT mice are currently considered the gold standard for HIV-1 research in murine models (17, 49, 50). BLT mice have been shown repeatedly to sustain mucosal HIV-1 infection and CD4+ T cell reconstitution in the FRT (32, 87).

The NSG-BLT mice have been frequently used for testing HIV-1 prevention and therapy. Studies examining therapeutics such as the long-acting ART raltegravir (88), ultra-long-acting antiretroviral dolutegravir (89), and PreP therapies such as the nucleoside reverse transcriptase inhibitor (NRTI) 4’-ethynyl-2-fluoro-2’-deoxyadenosine (EFdA) take advantage of the reconstituted human immune cell population in the mucosa (90). These studies have demonstrated effective inhibition of HIV-1 replication, reduction of HIV-1 viral load, and protection from multiple high-dose HIV-1 challenges (87, 88, 89, 90). Other studies using the NSG-BLT model provided valuable insights into HIV-1 treatment, viral evolution, prevention strategies, dose testing, tissue concentration, and pharmacokinetic data (74, 91–95). NSG-BLT Hu-mice have also been used to investigate potential treatment methods including anti–human IFN receptor 2 (IFNR2) (96) and anti-IFN-α/β receptor (IFNAR) antibodies (78) in conjunction with ARTs to successfully diminish viral reservoir size in lymphoid tissue and delay viral rebound (78, 96). A novel therapeutic strategy using chimeric antigen receptor modified stem cells successfully repopulated NSG-BLT mice with HIV-specific lymphoid populations and demonstrates potential for use in HIV treatment and cure studies (97). The efficacy of both HIV-1 reverse transcriptase inhibitor EFdA (98) and latency-reversing agents such as panobinostat (99) were also studied within the lymphoid compartments to elucidate effects on viral reservoir and latency. Finally, the NSG-BLT model is among the Hu-mice that can be used to evaluate the efficacy of potential HIV vaccines as demonstrated through significant T cell protection upon gag-specific vaccine administration (100). The development of proof of concept vaccines for therapeutic treatment has also been tested in the NSG-BLT model. In a lentiviral-based DC vaccine, HIV-1 antigen (SL9 epitope) is expressed with CD40 ligand to stimulate DC responses and Programmed Death 1 (PD-1) to prevent checkpoint activation (101). This vaccine demonstrated the ability to induce antigen-specific T cells and memory (101). Although unable to induce protection it was able to decrease viral load in the short term (101). In a similar model (NRG with fetal thymus implanted), another therapeutic vaccine expressing 5 CD4 and CD8 HIV specific T cell epitopes with CD40 ligand and administered with TLR3 agonist PolyI:C was successful at inducing anti-HIV CD8 and CD4 T cell responses, reactivated HIV reservoirs in cART controlled HIV infected mice, and decreased cell associated viral DNA (102).

## Understanding Mucosal Transmission of HIV-1 and the Effect of Microbiota Using Hu-Mouse Models

It is well recognized that more than 80% of HIV-1 infections occur through sexual transmission at mucosal surfaces, primarily the lower intestinal tract and female and male genital tract (8). While significant progress has been made in the understanding of mucosal transmission and pathogenic progression of HIV-1 through clinical studies and NHP models, the Hu-mice models present excellent model systems to recapitulate many features of mucosal infection in humans (103–106).

Most Hu-mouse experiments that have focused on mucosal (intrarectal or intravaginal) HIV-1 transmission have assessed prevention of infection using a wide variety of potential prophylactic agents. In these experiments, cell-free (including transmitted/founder strains) and cell-associated HIV-1 were used to challenge Hu-mice *via* the rectal or vaginal routes (24, 33, 64, 65, 94). While most of the mucosal prevention studies focused on the vaginal route of transmission, several have assessed the efficacy of PrEP preventions in Hu-mice challenged intrarectally. Topical microbicides (91), C5A in BLT mice (107), topically delivered ARVs (tenofovir disoproxil fumarate (TDF) and emtricitabine (FTC)) in BLT (108) are some of the prophylactic agents tested. Many studies tested various formulations, routes, pharmacokinetic and challenge routes. Different studies reported complete or partial protection against a single dose intrarectal challenge with HIV-1 (91, 94). Using the DKO model, tissue distribution of the interventions has also been assessed (73, 74). In studies focused on the vaginal route of transmission, many studies examined the efficacy of topical microbicides in DKO and BLT models (75, 92, 108–116). While most studies reported complete protection against single dose intravaginal challenge with HIV-1, others report only partial (89, 110, 113–117), or no protection (110).

Repeated, and often high dose, intravaginal exposure model has been tested to examine the effectiveness of the prophylactic intervention (88, 89, 94, 114). While it is likely that repeated, low dose viral challenges mimic vaginal transmission of HIV-1 in women more closely than high dose challenges, both experimental designs provide the opportunity to answer different research questions about prophylactic interventions. Interestingly, a few studies found delayed HIV-1 infection and dissemination when vaginal and systemic levels of drug were reduced or after drug cessation (112, 114, 115). Thus Hu-mice models can be useful in studying imperfect patient adherence and how this might impact HIV-1 transmission.

Work done by our group has highlighted the critical factors for successful mucosal transmission using a Hu-mouse model. We demonstrated that the frequency of circulating human CD45+ cells was the primary determinant of successful HIV-1 infection following intravaginal exposure in HSC-engrafted NRG mice. Furthermore, a significant correlation existed between peripheral blood CD45+ cells and HIV-1 target cells in the vaginal mucosa (64). This study highlighted that for successful HIV-1 infection through the intravaginal route, access to target cells in the mucosa is required. This highlights the importance of developing prophylactic interventions that limit target cells in mucosa, such as limiting tissue inflammation (118), to prevent HIV-1 infection.

The role of the microbiota in altering HIV susceptibility is a growing area of interest and the subject of many clinical studies. Hu-mice might be a useful model to examine the effect of the microbiota (vaginal and/or rectal) on HIV-1 acquisition, as a diverse vaginal microbiota low in *Lactobacillus* species is associated with a 4-fold increased risk of acquisition in women (119). If the next generation of Hu-mouse models engrafted with HSCs could be developed as gnotobiotic (germ-free) mice, this would allow for the reconstitution of Hu-mouse vagina/rectum/gut with human microbiota and assessment of HIV-1 acquisition risk. Although germ-free Hu-mice are not presently commercially available, a recent publication reported the generation of “pseudo-gnotobiotic” Hu-mice. NSG-BLT Hu-mice were treated with broad spectrum antibiotics, and subsequently transplanted with a human gut microbiota *via* fecal transplant; generating NSG-BLT mice reconstituted with human immune cells and a human gut microbiota. The authors found unique gut microbiota signatures in the mice that resembled those of the human donor, and they demonstrated that the human-like gut microbiota was stable in these mice for the duration of their study (14.5 weeks) (120). However, the relevance of this type of model and of other types of doubly-reconstituted Hu-mice (immune cells and microbiota) that we may be able to generate in the future is controversial at present. This is for a variety of reasons including, but not limited to, our lack of knowledge on generalizability of results obtained in mice to humans, a lack of standardized protocols, inter-donor, ethnic, and geographical microbial variability that makes replication of data challenging, and anatomical and physiological differences between mice and humans that might impact the microbiota (121). Nevertheless, germ-free humanized mouse models that can be reconstituted with a human-like microbiota may one day be key advancements that improve our understanding of the role of the vaginal, rectal and gut microbiota in mucosal HIV-1 transmission, epithelial barrier disruption, and inflammation. Furthermore, they may be useful in examining prophylactic interventions to decrease systemic inflammation and prevent HIV-1 transmission.

## Using Hu-Mice for Understanding Tuberculosis-HIV Co-Infection

Currently animal models for HIV co-infection with other pathogens are lacking. Although Hu-mice have been used to investigate HIV co-infection with pathogens such as Epstein–Barr virus and *Neisseria gonorrhoeae* (30, 122), co-infection with *Mycobacterium tuberculosis* (Mtb) is of particular interest as it is the most common cause of AIDS-related death (1). HIV-1 infection increases the risk of latent tuberculosis (TB) reactivation (123). HIV/TB co-infection increases morbidity and mortality while complicating therapies associated with both diseases due to multiple factors including the development of Immune Reconstitution Inflammatory Syndrome (IRIS) and TB drug resistance (124). The current inbred mouse *in vivo* models of TB do not develop organized granulomas (125) and show inconsistent immune responses (126).

On the other hand, the use of Hu-mouse models (HSC-engrafted (127, 128) and BLT-engrafted (129) NSG mice) has demonstrated tremendous potential to recapitulate human TB infection, immune response, and formation of organized granulomas (127–129). With the vastly successful Hu-mice studies in HIV-1, they serve as a viable model for co-infection. In early HIV/TB co-infection studies, NSG-BLT mice were infected with HIV-1 followed by Mtb. HIV-1 was localized in pulmonary granulomas and exacerbated TB lesions and lung pathology were seen (130). A more recent study demonstrated that the same model can be used for studying TB relapse in co-infection by administration of HIV-1 intravenously after paucibacillary TB infection was established (131). Although only NSG-BLT model has been used thus far for TB-HIV co-infection, newer generation Hu-mouse models using the easier and more accessible HSC-only engraftment method for HIV/Mtb co-infection would allow for more widespread use of the model, thus addressing the lack of literature on HIV/TB co-infection studies *in vivo*.

## Improved Hu-mouse Models for HIV-1 Research (Next Generation): Nsg-A2, DRAG, DRAGA, and BRGST

### Addressing the Challenges With the BLT Model

Even though the BLT model is currently the gold standard for HIV-1 research, there are several disadvantages that limit its use. Xenogenic GvHD that develops post-engraftment (132, 133) remains a concern despite efforts to extend longevity using a triple-knockout model (134, 135). This reduces the sample population of mice in studies (17) and prevents long-term studies. Humanized BLT mice also lack high levels of B cell populations and hyper-mutated, class-switched IgG antibodies (136). Furthermore, the engraftment of human fetal liver and thymus tissue is time-consuming and requires great technical skill to execute. Finally, a major issue with using the fetal BLT method is material availability, as restrictions on the use of fetal tissue in research is of increasing concern (137). To address this shortcoming, a novel method of using neonatal thymus tissue to replace the use of fetal tissue was developed within NOD,B6.SCIDIl2rg^-/-^Kit^W41/W41^ (NSGW) mice (NSGW-NeoThy) (39, 138). The addition of the *Kit^W41/W41^* alleles offers the advantage of accepting HSC engraftment without prior irradiation (138, 139). Neonatal thymic samples are easier to obtain, and yield much larger quantities of tissue and can thus humanize more mice per sample compared to using fetal tissue (39). NSGW-NeoThy mice developed smaller thymic organoids but with either autologous or allogeneic HSC engraftment, the model successfully repopulated human myeloid and lymphoid populations comparable to fetal thymus-only engrafted NSG mice (39), thus demonstrating its potential for future use in HIV investigations. Some evidence also presented the potential of reduced GvHD in NSGW-NeoThy mice by administration of anti-human CD2 antibodies to remove GVHD-associated passenger thymocytes, but a more comprehensive study must be conducted to elucidate GvHD development in the model (39).

### Next Generation of Transgenic Mice

NSG-A2 mice were developed from the NSG background strain and are transgenic for the human HLA class I-A2 molecule. When humanized with HLA-matched HSCs, this allows human CD8+ T cells to be functionally mature (140). However, neither total CD8+ T cell reconstitution levels nor B cell function were significantly better than NSG mice (140, 141). To improve the humoral immune response, the HLA class II transgene (specifically, HLA-DR4) molecule has been expressed in the NOG (142), NSG (143), and NRG mice (34). Here we are focusing on the more popularly used and radiation-tolerant NRG background termed DRAG mice.

Humanized DRAG (hu-DRAG) mice with HSC derived HLA-DR-matched umbilical cord blood engraftment resulted in significantly higher counts of human CD4+ and CD8+ T cells compared to its non-transgenic NRG counterpart (34) (**Table 1**). Human B cells were highly functional, and could undergo immunoglobulin (Ig) isotype class-switching (34). To adequately compare the benefits between transgenic HLA class I and II, a model co-expressing both the HLA-A2 and HLA-DR4 molecules, termed DRAGA mice was developed (35). Comparisons between NRG-A2, DRAG, and DRAGA models engrafted with HLA-matched HSCs demonstrated that both hu-DRAG and hu-DRAGA models had significantly better human T-cell reconstitution, CD4/CD8+ T cell function, and most importantly, significant B cell Ig class-switching when compared to NRG-A2 mice (35) and even the hu-BLT models (136) (**Table 1**). These results demonstrate that the HLA-DR4 transgene can confer more benefits in human lymphoid reconstitution compared to HLA-A2.

Recently the BRG background was altered to produce a promising model with consistent lymph node reconstitution and development addressing the shortcomings of secondary lymphoid tissue formation within current Hu-mouse models (37, 144). Balb/c *Rag2* ^−/−^ *Il2rg* ^−/−^ *Sirpa* ^NOD^ (BRGS) mice (145) that express transgenic thymic-stromal-cell-derived lymphopoietin (TSLP), termed the BRGST model, boast robust human cellular and humoral responses (37). TSLP is similar in structure and function to IL-7, but is IL2rg independent and thus can promote B and T cell responses (39). In particular, when compared to the older hu-BRGS model, hu-BRGST mice demonstrated enhanced Ig-isotype class switching, central/effector memory T cell, and T follicular helper (TFH) cell development in secondary lymphoid tissues with pronounced B cell zones (37). When the BRGS background hosts HLA class I and II transgenes (termed BRGSA2DR2 mice), improvements in T and B cell development and functionality including Ig-isotype class switching and antigen-specific responses were also observed (38).

Hu-DRAG mice are capable of supporting HIV-1 infections when challenged intravaginally as the mucosa of the FRT and gut both repopulate with CD4+ T cells and TFH cells (36, 146). Hu-BRGST mice successfully sustain HIV-1 infection and replication upon intraperitoneal inoculation (37). Viral reservoir and latency was also demonstrated after HAART administration, thus also offering possibilities in HIV latency and cure investigations (37). As hu-DRAG, hu-DRAGA, hu-BRGST, and hu-BRGSA2DR2 mice develop robust antigen-specific Ig responses, these models have tremendous potential for use in testing novel HIV-1 vaccine formulations. Immunization of both hu-DRAG and hu-DRAGA models for the investigation of other pathogenic viruses such as influenza (35, 147, 148) and Zika (149) have already yielded promising results (34, 35, 146–149). Therefore, the hu-DRAG and hu-DRAGA demonstrate tremendous potential for future use in HIV-1 therapeutic antibody and vaccine research.

### Other Novel Models for HIV-1 Studies

The reconstituted human immune cell population in the current HSC-engrafted models for HIV-1 studies consist mainly of lymphoid cells with lower overall functional NK cell and myeloid repopulation (42, 150–152). Reduced myeloid populations may result in decreased endogenous cytokine signals, preventing the model from providing the full human inflammation process (42). Additionally, this may limit aspects of HIV-1 investigation such as innate immunity, antigen presentation interactions, or humoral immunity and vaccine studies. **Table 1** summarizes some of the novel models including, MITRG/MISTRG models (discontinued by the Jackson Laboratory- short life span of 10-16 weeks post engraftment), NSGS (also called NSG-SGM3) model (NSG mice expressing human myeloid promoting cytokines SCF, GM-CSF, and IL-3, life span issue after 20 weeks) (42–44), and NOG-EXL (NOG mice expressing GM-CSF and IL-3) (40, 41) for better human myeloid cell engraftment of monocytes/macrophages and NK cells reconstitution (46, 47). Furthermore, the NSG-15 (45) and SRG-15 (31) models have been developed to express transgenic human IL-15 specifically for improved NK cell development. Overall, these models have all demonstrated success in their use for HIV-1 investigations, and their myeloid and NK reconstitution improvements can further extend HIV-1 *in vivo* research capabilities. It is important for researchers to note that until all shortcomings of Hu-mouse models have been addressed, choosing the optimal model for a study will depend on the experiment itself with special considerations for study timeline and immune cells of interest.

## Conclusion

In summary, the development of Hu-mouse models has provided a cost-effective and practical approach for HIV-1 research. These mice provide a useful pre-clinical tool, since they allow researchers to directly examine interactions between HIV-1 and the human immune system. Novel modifications in generating Hu-mice is increasing the feasibility of using these models to investigate more complex clinical problems, such as immune response in co-infections like HIV and TB, and understanding interactions between immune responses and microbiota in regulating HIV-1 susceptibility. As we continue to make improvements in humanization of mice by developing novel models with new features and gain better understanding of how to tailor the models to answer specific questions, we will continue to push the envelope and make breakthroughs in HIV-1 research.

## Author Contributions

AG and CK outlined the content of the review. JY and JW prepared the main body of the manuscript. AG and CK revised the manuscript. AG supervised the project. All authors contributed to the article and approved the submitted version.

## Funding

CK’s laboratory is funded by the Canadian Institutes of Health Research Operating Grant (FRN# SOP 159229). Salary support (JW) is provided by a Canadian Institutes of Health Research Postdoctoral Fellowship award (MFE 152502).

## Conflict of Interest

The authors declare that the research was conducted in the absence of any commercial or financial relationships that could be construed as a potential conflict of interest.
